# Integration of Imprint-Free and Low Coercivity Ferroelectric BaTiO_3_ Thin Films on Silicon

**DOI:** 10.1021/acs.nanolett.5c05139

**Published:** 2026-01-08

**Authors:** Jingtian Zhao, Majid Ahmadi, Beatriz Noheda, Martin F. Sarott

**Affiliations:** † Zernike Institute for Advanced Materials, 3647 University of Groningen, 9747AG Groningen, The Netherlands; ‡ Groningen Cognitive Systems and Materials Center (CogniGron), University of Groningen, 9747AG Groningen, The Netherlands

**Keywords:** ferroelectrics, oxide thin films, epitaxy, BaTiO_3_, silicon-compatible, imprint-free

## Abstract

Highly crystalline ferroelectric oxides integrated on Si hold great promise for energy-efficient memory and logic technologies. Exploiting epitaxial strain engineering in these materials is, however, severely hampered on Si, where the large structural mismatch often results in an inferior interfacial quality and causes degradation of the ferroelectric switching characteristics. In this work, we present the growth of single-crystalline BaTiO_3_ thin films on Si, exhibiting imprint-free switching, low coercivity, high remanent polarization, and no fatigue for over 10^10^ switching cycles. We accomplish this via the insertion of a SrSn_1–*x*_Ti_*x*_O_3_ layer on SrTiO_3_-buffered Si. This layer serves as a pseudosubstrate that alleviates the thermal strain that the Si substrate imposes on the BaTiO_3_ layer, while simultaneously providing moderate compressive strain that stabilizes a pure out-of-plane polarization. Thus, our work paves the way toward the fabrication of Si-compatible, low-power-consuming ferroelectric devices.

## Graphical abstract

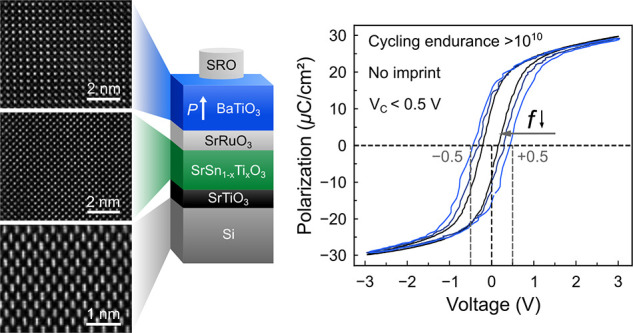

The growing demand for energy-efficient memory and logic devices drives the need to integrate new materials with additional functional properties into the complementary metal-oxide semiconductor (CMOS) platform.^1^ In this regard, ferroelectric materials, which uniquely feature spontaneous electric polarization switchable by an external electric field, are particularly attractive. When prepared in the form of highly crystalline epitaxial thin films, it is possible to finely tune the ferroelectric domain configuration, the direction and magnitude of the polarization, and the resulting macroscopic electrical properties of ferroelectric oxides, utilizing the epitaxial strain imposed by single-crystalline substrates.^2–6^ In CMOS, due to the substrate being restricted to silicon, the use of strain engineering is not applicable in general, which obstructs the integration of optimized ferroelectric thin films into Si-based heterostructures. Hence, rendering complex oxides, especially ferroelectrics, compatible with silicon has remained a major challenge.

Among the various ferroelectrics, growing the perovskite-structure material BaTiO_3_ (BTO) on silicon has attracted a lot of interest due to its robust ferroelectric properties in the thin-film regime, large dielectric constant, and absence of volatile or toxic elements. Several attempts have been made to integrate BTO on silicon substrates for diverse applications, including nonvolatile memory,^7^ ferroelectric tunnel junctions (FTJs),^8–10^ and electro-optic devices.^11–13^ For these applications, the fabrication of BTO thin films on silicon has been accomplished using a number of deposition methods, including atomic layer deposition (ALD),^14^ molecular beam epitaxy (MBE),^15,16^ radio frequency magnetron sputtering,^17^ and pulsed laser deposition (PLD).^18–22^ A common difficulty that persists when attempting to grow high-quality BTO, as well as other complex oxides, on silicon, independent of the deposition method, is the large structural mismatch between the oxide and silicon. To address this issue, the use of buffer layers, such as SrTiO_3_ (STO),^14,20^ yttria-stabilized zirconia,^23^ and other perovskites^19^ has been a popular strategy to both enable a controlled growth of BTO with varying thickness and tune the ferroelectric properties via strain engineering.^23^ This, hence, showcases that lattice matching using suitable buffer layers might be an auspicious route to optimize the properties of BTO thin films on silicon.

Despite many promising recent developments, even with the help of buffer layers, an additional complication arises due to the large mismatch in the coefficients of thermal expansion (CTE) between Si and oxides^18^ (Si: ∼2.6 × 10^–6^ K^–1^ vs SrTiO_3_: ∼8.7 × 10^–6^ K^–1^). During cooldown from the elevated growth temperatures of oxides, this CTE mismatch introduces significant *thermal strain*,^18,24,25^ which can lead to extended structural defects. In ferroelectrics, this thermal tension can further trigger the uncontrolled formation of domains,^26^ which, in turn, can detrimentally impact the macroscopic electric-field response. Furthermore, for BTO thin films on silicon, due to the tensile nature of the thermal strain, it has hitherto remained difficult to maintain a large remanent out-of-plane polarization with a low coercive field (<1 V), while simultaneously minimizing imprint. Imprint refers to a built-in bias in a ferroelectric heterostructure that leads to a preferential direction of a polarization and manifests as a horizontal shift in the hysteresis loop.^27^ In extreme cases, imprint can entirely destabilize one polarization direction in the absence of an electric field (i.e., at remanence) and render a ferroelectric unipolar, such that it can only be switched in a volatile manner. Generally, imprint can originate from multiple sources with the most common one in epitaxial thin films being the presence of asymmetric top and bottom electrodes with different work functions.^28,29^ Additionally, inhomogeneous epitaxial strain, defect dipoles, and surface defects^27^ can also significantly contribute to imprint and hamper the realization of reliable and truly nonvolatile memory devices.^27^

In this work, we present imprint-free BTO thin films on silicon substrates with a low coercivity, a high remanent polarization, and an excellent fatigue resilience (>10^10^ cycles). We achieve this by introducing a strain-mediating layer of SrSn_1–*x*_Ti_*x*_O_3_ (SSTO) acting as a pseudosubstrate on STO-buffered Si substrates. By purposefully relaxing this SSTO layer, we minimize the thermal tension experienced by the BTO layer and simultaneously mimic on silicon the moderate compressive epitaxial constraints of the commonly used scandate substrate material GdScO_3_ (GSO). Remarkably, our BTO films grown by pulsed laser deposition exhibit extremely low leakage, enabling the fabrication of ultrathin BTO devices with robust ferroelectric properties. In conjunction with good polarization retention and high fatigue resistance, this work thus paves the way for Si-compatible ferroelectric devices such as ferroelectric field-effect transistors or FTJs for low-energy-consuming oxide electronics.

We start our investigation by optimizing the growth of the BTO-based heterostructure on TiO_2_-terminated STO (see Experimental Section) before moving to STO-buffered Si substrates. To do so, we first deposit a 60 nm thick layer of SrSn_0.45_Ti_0.55_O_3_ (hereafter referred to as SSTO), followed by a 10 nm thick SrRuO_3_ (SRO) bottom electrode, and a 30 nm BTO layer (see Figure 1a). For all layers, we monitor the film thickness *in situ* with unit-cell precision using reflection high-energy electron diffraction (RHEED) and confirm it *ex situ* via X-ray reflectivity. By tailoring the B-site cation ratio in SSTO, i.e., the Sn:Ti ratio, it is possible to linearly tune its lattice parameter to any value between 4.033 Å (pure SrSnO_3_) and 3.905 Å (pure SrTiO_3_).^30,31^ Here, in order to emulate the epitaxial constraints of GdScO_3_ (*a*_pc_ = 3.963 Å) with the SSTO pseudosubstrate layer, we select an SSTO composition of SrSn_0.45_Ti_0.55_O_3_. BTO films grown under moderate compressive strain on GdScO_3_ (−0.9%) have been shown to exhibit a robust out-of-plane polarization that persists up to large film thicknesses without the formation of in-plane polarized *a*-domains.^20,32,33^ Hence, by purposefully letting the SSTO layer relax on STO, it should be possible to obtain BTO-based heterostructures with ideal elastic boundary conditions for out-of-plane ferroelectricity on silicon while preserving (quasi-)­epitaxial quality.

**Figure 1:**
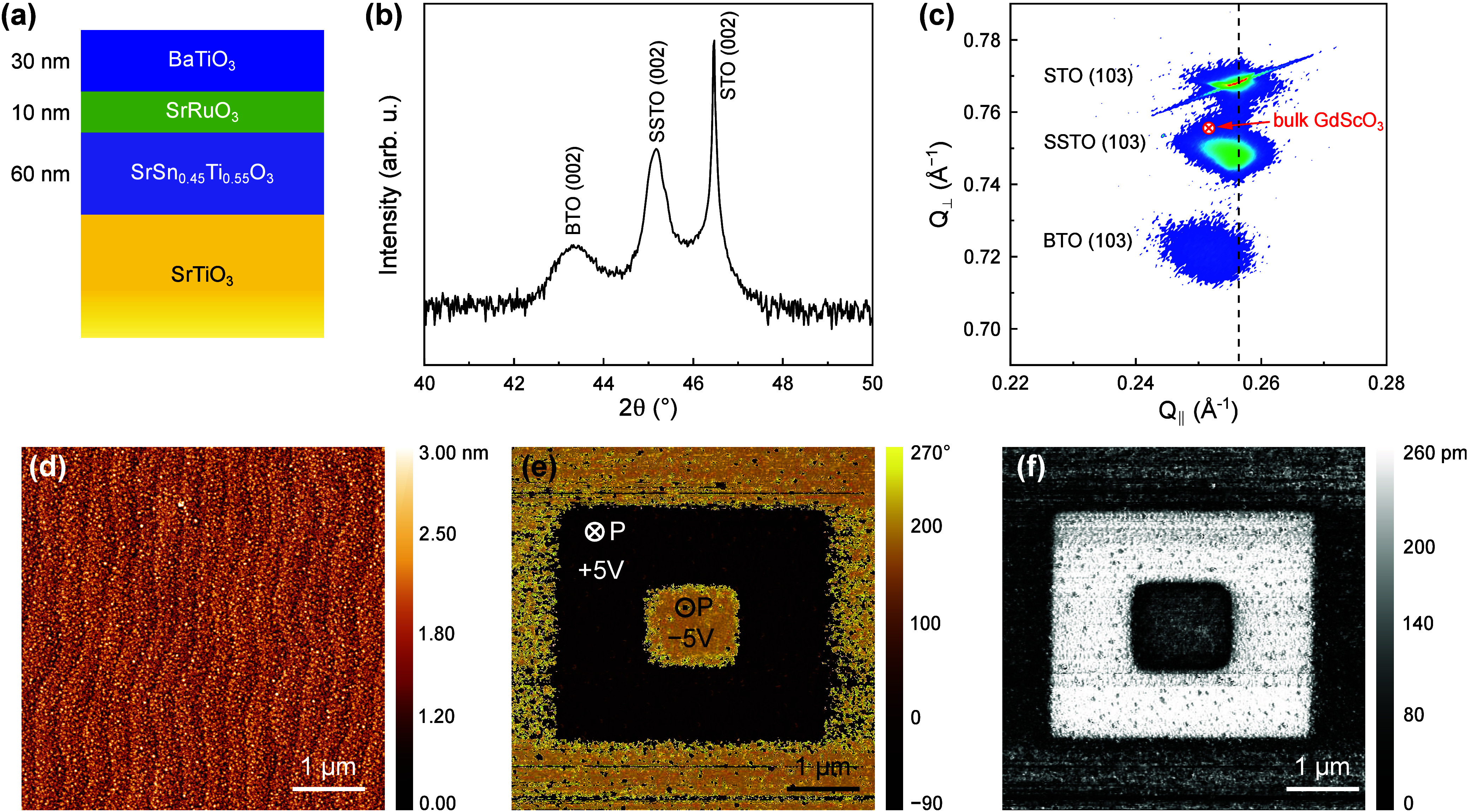
Structural and local ferroelectric characterization of BaTiO_3_ (BTO) on SrSn_0.45_Ti_0.55_O_3_ (SSTO)-buffered SrTiO_3_ (STO). (a) Illustration of the heterostructure BTO/SRO/SSTO grown on TiO_2_-terminated STO. (b) Symmetric XRD θ–2θ scan of the heterostructure around the STO 002 peak. (c) XRD reciprocal space map around the STO 103 reflection. (d) 5 × 5 μm^2^ atomic force microscopy topographic image. Vertical piezoresponse force microscopy (PFM) phase (e) and amplitude (f) responses across an electrically poled box-in-box region.

To characterize the out-of-plane crystallographic orientation of the epitaxial heterostructures, symmetric θ–2θ X-ray diffraction (XRD) measurements are performed, as shown in Figure 1b. Distinct 002 reflections from the BTO, SSTO, and STO layers are observed, indicating that all of the layers are well-aligned along the [001] direction. The absence of secondary phases or polycrystalline peaks confirms the high crystallinity of the films, and notably, we do not observe any evidence for *a*-domain formation that would give rise to BTO 200/020 reflections. The reciprocal space map (RSM) around the STO 103 peak, shown in Figure 1c, further reveals the epitaxial relationship among the layers in the heterostructure. Specifically, we observe that both the SSTO and the BTO layers are partially relaxed from their respective underlayer, i.e., the STO and the SSTO, respectively. The atomic force microscopy (AFM) image in Figure 1d shows that the BTO surface is atomically flat without detectable islands (*R*_q_ < 4 Å) and clear step-edge terraces, pointing toward ideal two-dimensional layer-by-layer growth. The ferroelectric nature and local switching behavior of the BTO film is further confirmed by piezoresponse force microscopy (PFM). Figure 1e and Figure 1f display the vertical PFM phase and amplitude images, respectively, recorded after applying electrical biases of ±5 V to the PFM tip in a box-in-box pattern. The phase image clearly exhibits a 180° reversal of polarization upon the application of opposite biases. Furthermore, the amplitude exhibits a clear minimum at the location of the 180° domain walls (Figure S1). In brief, the introduction of the SSTO buffer layer as a pseudosubstrate effectively reduces the lattice mismatch of the BTO film with the STO substrate, resulting in a purely out-of-plane polarized BTO film that is reversibly switchable.

Next, to check whether this buffer-layer strategy indeed enables us to maintain the high structural quality and the single-domain configuration of the BTO film on Si, we prepared a series of BTO films with varying thicknesses by PLD on STO-buffered Si (001) substrates (see Figures 2a and S2). For simplicity, we focus our discussion here on a representative sample with a BTO thickness of 30 nm, while the thicknesses of the underlying SSTO and SRO layers are kept constant at 60 and 10 nm, respectively. The specular XRD θ–2θ scan of this heterostructure, shown in Figure 2a, shows clear 00l reflections of BTO and SSTO and confirms the preservation of the *c*-axis-oriented growth of BTO on silicon (see also Figure S3). Again, no *a*-domains (2θ_200/020_ = 45.3°) or secondary phases are detected and the films are purely *c*-oriented (see also the symmetric RSM around BTO 002 in Figure S3), analogous to the BTO-based heterostructure grown on single-crystalline STO in Figure 1. Furthermore, as shown in the inset of Figure 2a, during BTO growth, we observe pronounced RHEED intensity oscillations, which indicate near-ideal layer-by-layer growth and are consistent with the atomically flat AFM surface topography (see Figure S4). While not being essential for achieving high-quality BTO films on STO-buffered Si substrates,^20^ having *in situ* thickness control with unit-cell precision is important for the realization of ultrathin ferroelectric tunneling-based devices and confirms the optimization of the growth parameters.

**Figure 2:**
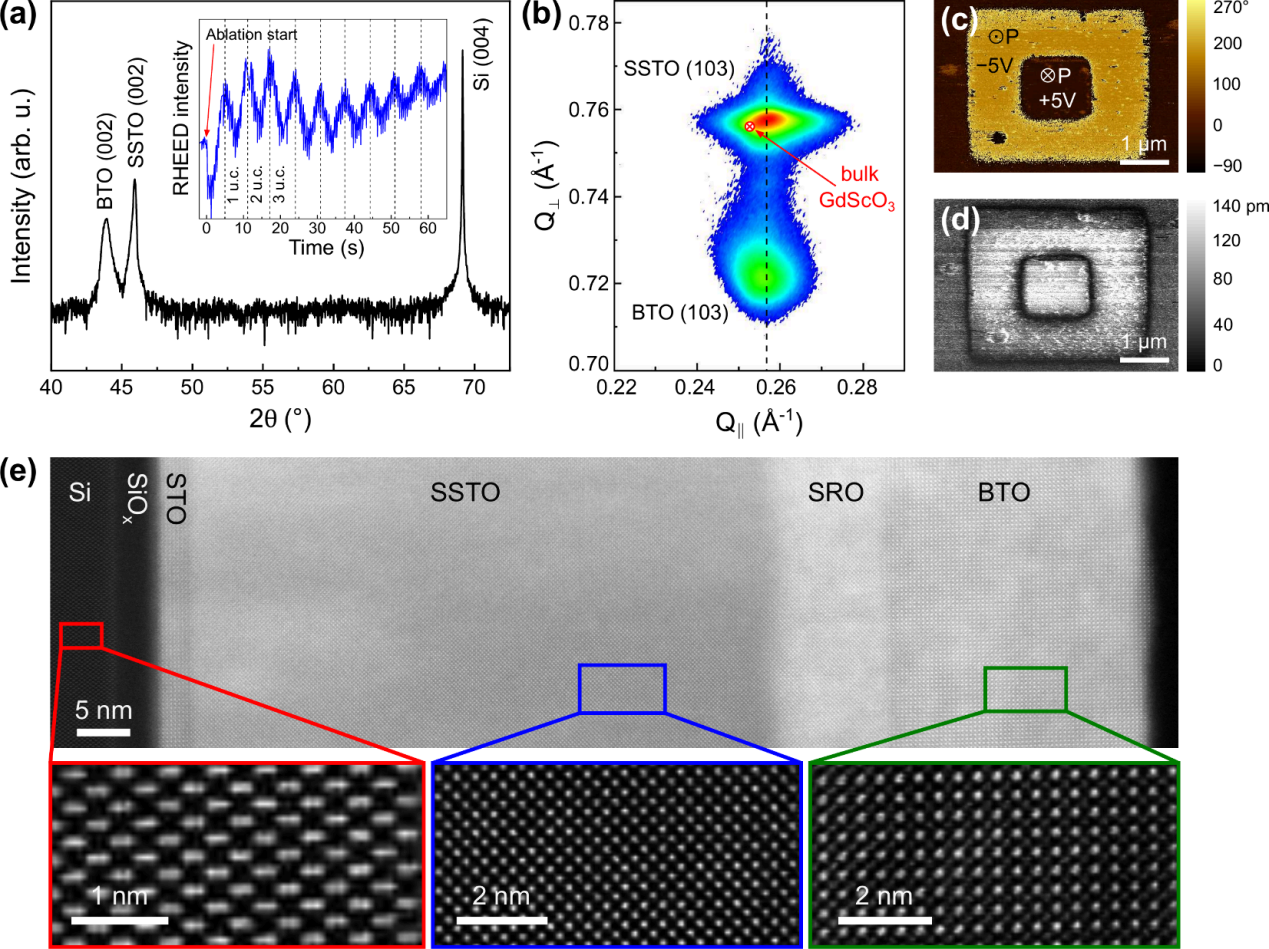
Structural and local ferroelectric characterization of BTO on Si. (a) Symmetric XRD θ–2θ scan of the heterostructure with the inset showing the *in situ* acquired integrated RHEED time trace during BTO growth. (b) XRD reciprocal space map around the SSTO 103 reflection, showing that the SSTO lattice parameters are close to those of GdScO_3_ (known to provide optimal strain conditions for out-of-plane polarized BTO). (c,d) Vertical PFM phase (c) and amplitude (d) responses across an electrically poled box-in-box region. (e) Cross-sectional HAADF-STEM image of the BTO heterostructure with magnified views of the SSTO and BTO layers and the Si substrate.

In contrast to the BTO grown on bulk STO, the RSM around the SSTO 103 reflection on STO-buffered Si, shown in Figure 2b, reveals a fully relaxed state of the SSTO with its lattice constants lying very close to that of bulk GdScO_3_, whereas the BTO film becomes fully strained to the underlying SSTO. The complete strain relaxation of the SSTO in this case can be likely attributed to the compressive strain state of the thin STO buffer layer on Si (001) compared to bulk STO,^34^ which increases the lattice mismatch between STO and SSTO and promotes the relaxation of the latter. Cross-sectional scanning transmission electron microscopy (STEM) measurements, shown in Figures 2e and S5, further highlight the crystalline quality of the films, the absence of structural defects, and the 45° rotation of the perovskite layers with respect to the Si unit cell. Hence, despite using different substrates, the θ–2θ scans, RSMs, rocking curves, and STEM images in Figures 1, 2, S3, and S5 showcase the crystallinity and the out-of-plane orientation of the BTO/SRO/SSTO heterostructures on Si. We attribute this to the role of the SSTO layer, which successfully mimics the epitaxial constraints of the GdScO_3_ substrate and, therefore, effectively decouples the BTO layer from the mechanical boundary conditions of the substrate.

The vertical PFM phase (Figure 2c) and amplitude (Figure 2d) images after DC poling using the PFM tip further reveal reversible out-of-plane ferroelectric switching, comparable to that of BTO on STO. Notably, the pristine out-of-plane orientation of the BTO polarization on STO-buffered Si is predominantly downward and, thus, opposite to the upward-oriented polarization observed in the BTO films grown on STO. This difference may originate either from variations in substrate/interface termination, defect chemistry, or the minor difference in the epitaxial boundary conditions.^5,35–37^ Importantly, in comparison to previous studies that have reported strong depolarization effects in BTO films on STO-buffered Si, which cause spontaneous back-switching of poled domains within 10 min,^20,26^ our films maintain a clear PFM contrast for at least 100 min (see Figure S6). This enhanced polarization stability underscores the improved structural and chemical homogeneity of our BTO films via a reduction of thermal stresses and improved lattice matching enabled by the SSTO buffer.

Realistic ferroelectric-based devices require controlled and repeatable polarization switching in a parallel-plate geometry. To investigate the macroscopic ferroelectric properties of our BTO heterostructures, we therefore fabricate BTO capacitor structures with symmetric SRO electrodes (i.e., SRO/BTO/SRO/SSTO on STO-Si). Figure 3a shows the *P*-*E* hysteresis loops of a 30 nm thick BTO capacitor, measured at a frequency of 5 kHz across a range of voltages. Upon increasing voltage amplitude, the *P*-*E* loops become well-saturated with remanent polarization (*P*_r_) values of approximately 20 μC cm^–2^, which closely aligns with the values reported for bulk BTO and comparable to those observed in BTO thin films on oxide substrates.^38^ Most notably, in comparison to previous reports of BTO films on Si,^19,20^ the BTO films here exhibit a lower coercive voltage of 0.61 V (203.76 kV cm^–1^), while being essentially free of imprint $\frac{V_{c,+}+V_{c,-}}{2}=0.04\pm 0.03\;V$ . The absence of imprint indicates that the insertion of the SSTO buffer is highly effective against the formation of built-in fields that can pin the ferroelectric polarization. This includes previously reported structural causes of imprint, such as strain gradients due to thermal strain or defect dipoles^39,40^ that are commonly observed even when employing symmetric electrodes. In addition to the lack of imprint, the corresponding current–electric field (*I*-*E*) profiles (Figure 3b) show a remarkable absence of leakage, even for applied electric fields (>2 MV cm^–1^) that by far exceed the coercivity.

**Figure 3:**
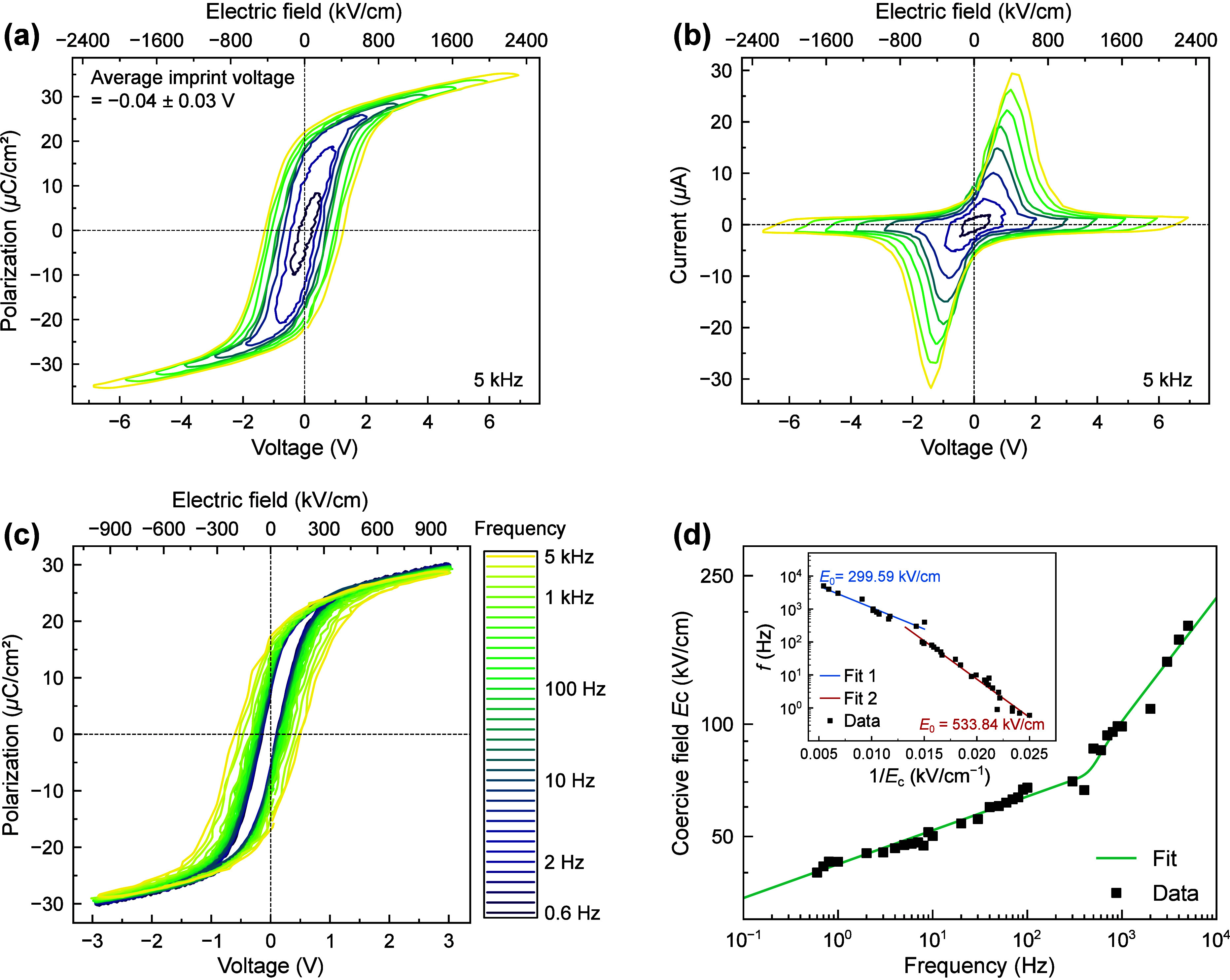
Macroscopic ferroelectric characterization of BTO on Si. (a) Polarization–electric field hysteresis loops measured at 5 kHz for various electric field amplitudes, showing lack of imprint. (b) Switching current profiles corresponding to the loops in (a). (c) Frequency-dependent hysteresis loops over the range 0.6 Hz to 5 kHz showing coercive voltages (*V*_c_) in the range of 0.12–0.55 V. (d) Coercive field (*E*_c_) as a function of frequency extracted from (c). The inset shows the Merz-law fitting of the same data set.

To further deepen our understanding of the BTO switching dynamics, we investigate the frequency dependence of the hysteresis loops. Figure 3c contains the BTO *P*-*E* loops over a frequency range from 0.6 Hz to 5 kHz with a fixed maximum applied voltage of 3 V. For all measured frequencies, the BTO films remain free of leakage and imprint, but we observe a clear reduction of the coercive voltage with decreasing frequency from 0.55 V at 5 kHz to 0.12 V at 0.6 Hz. This is, in fact, consistent with reports in many ferroelectric bulk crystals and thin films, where higher frequencies are typically accompanied by a notable increase in the coercive field.^41–44^ Within the theoretical Ishibashi–Orihara framework based on the Avrami model,^42,45,46^ the frequency dependence of the coercive field follows a power law *E*_c_ ∝ *f* ^β^. For the BTO films considered here, however, the log­(*E*_c_) vs log­(*f*) plot, shown in Figure 3d, clearly exhibits two distinct linear scaling regimes with a crossover at around 500 Hz that cannot be fitted with a single power-law exponent β. This, hence, implies two distinct types of switching kinetics contributing to polarization reversal, as observed similarly for bulk relaxor PMN–PT crystals^42^ and PZT thin films.^28,41,43^ Taking into account two distinct switching kinetics regimes and using an empirical expression proposed by Chen et al.,^42^ we are indeed able to fit our data.

Interestingly, our measured crossover frequency of ∼500 Hz lies close to that of 200–500 Hz reported for PZT thin films.^41^ This similarity points toward a common underlying mechanism for the observed change in domain-switching dynamics with thermally activated domain-wall creep dominating polarization switching at low frequencies and viscous domain-wall flow governing at high frequencies.^41,47^

Applying Merz’s law^48,49^ to the frequency dependence of *E*_c_

1 $$f=f_{0}\exp\left(-\frac{E_{0}}{E_{c}}\right)$$

allows us to quantify the activation fields *E*_0_ of the high- and low-frequency switching regimes. To do so, we plot log­(*f*) vs (1/*E*_c_), as shown in the inset of Figure 3d, where we extract activation fields of 533.8 kV cm^–1^ in the low-frequency and 299.6 kV cm^–1^ in the high-frequency regime. For comparison, similar measurements in PMN–PT single crystals have yielded activation field values ranging between 20–70 kV cm^–1^,^42^ whereas in epitaxial oxide thin films activation fields have been found to lie broadly spread between 100 and >1000 kV cm^–1^.^50^

Dynamic hysteresis measurements, such as those in Figure 3, contain multiple current contributions from ferroelectric switching, capacitive charging, or leakage and therefore often lead to an overestimation of the intrinsic ferroelectric polarization. In addition, they fail to capture spontaneous back-switching due to depolarization. To assess the true intrinsic remanent polarization of our films, we perform positive-up-negative-down (PUND) measurements, as shown in Figure 4a, for a 30 nm thick BTO capacitor. The obtained 2*P*_r_ value of 16 μC cm^–2^ indicates that our BTO films on Si exhibit a strong intrinsic polarization after eliminating all non-ferroelectric current contributions. This polarization further remains robust under electric field cycling without any noticeable fatigue even after 10^10^ cycles (Figure 4b) and shows good device-level retention (Figure S7). Note that in comparison to previous studies of BTO on Si,^20^ our fatigue and retention measurements do not require biasing to compensate for device imprint. Hence, by getting rid of imprint in our BTO heterostructures via the introduction of the strain-mediating SSTO pseudosubstrate, we essentially eliminate fatigue, one of the most serious reliability concerns in ferroelectric films, and surpass previously reported BTO cycling endurance limits. This likely points to a superior interfacial quality and a suppressed influence of intrinsic defects, such as oxygen vacancies, on cycling performance.

**Figure 4:**
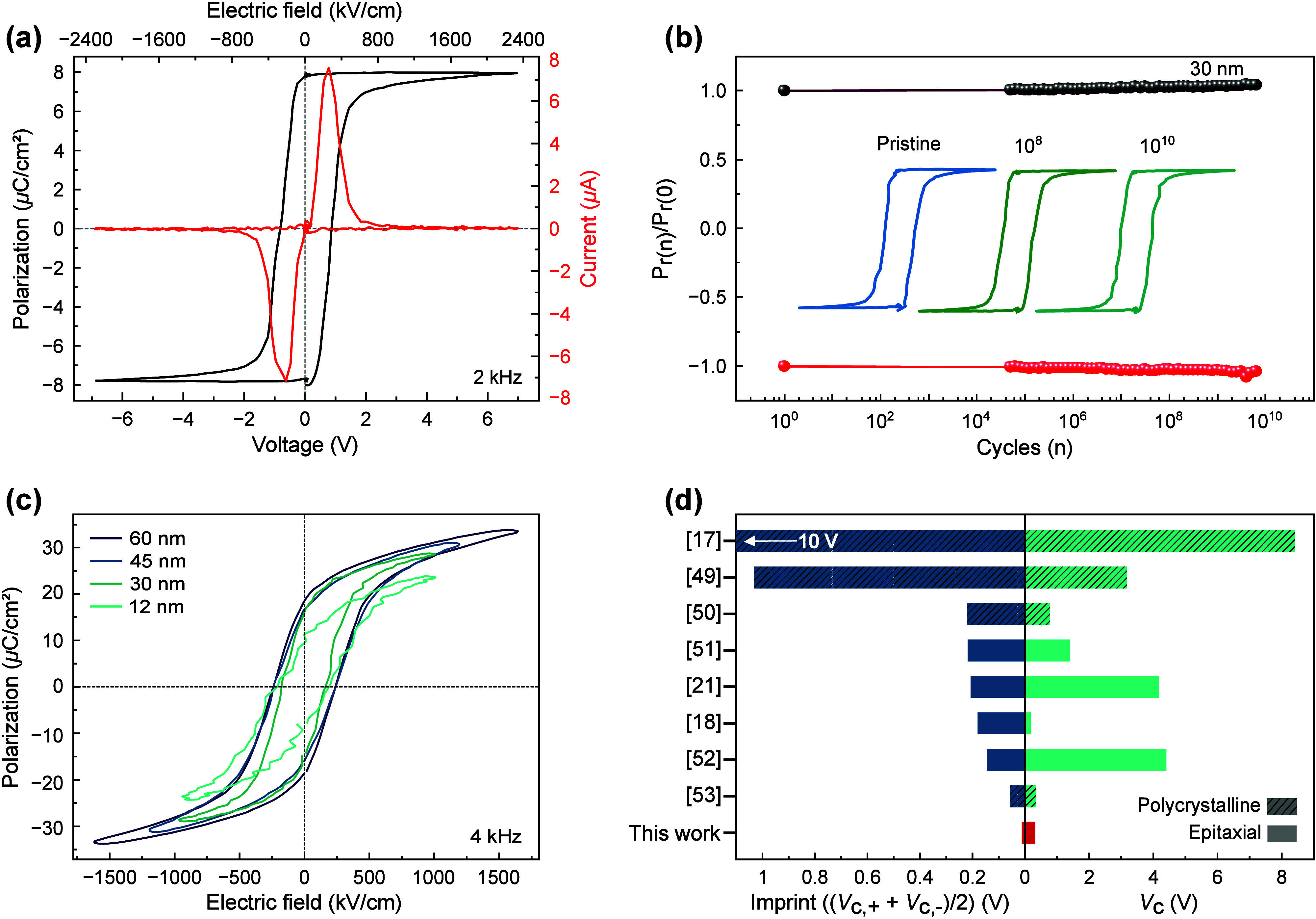
Robustness of the BTO switching characteristics. (a) PUND hysteresis loops and corresponding switching current profiles measured at 2 kHz. (b) Ferroelectric cycling endurance over 10^10^ cycles measured with a voltage amplitude of 4 V and a frequency of 2 kHz. The inset shows the PUND loops obtained after 1, 10^8^, and 10^10^ cycles. (c) Dynamic hysteresis loops for BTO films on Si with different thickness. (d) Literature comparison of the device imprint and *V*_c_ in BTO thin films on Si substrates, with the vertical axis indicating the reference numbers.

The robustness of the ferroelectric switching characteristics is also maintained when the thickness of the BTO layer is varied in the heterostructures. The dynamic hysteresis measurements in Figure 4c display saturated, symmetric, and imprint-free ferroelectric switching characteristics even when reducing the BTO thickness to 12 nm (see also Figures S8, S9, and S10). Interestingly, we observe that the coercive field remains largely independent of the BTO thickness (see Figure S10) for the entire thickness range up to *d* = 60 nm and, therefore, deviates from the Kay–Dunn scaling (*E*_C_ ∝ *d* ^–2/3^) at larger film thicknesses than previously reported.^51^ Furthermore, as summarized in Figure 4d and Table S1, a literature comparison of the imprint in BTO-based thin films on Si substrates shows that the imprint of our epitaxial BTO thin films is substantially lower than previously reported values.^19,20,23,52–56^ Achieving imprint-free ferroelectric devices on silicon constitutes a key step toward reliable long-term cycling and high-frequency operation and promotes their integration into advanced ferroelectric memory and logic devices.

In short, we demonstrate the epitaxial integration of BTO thin films on STO-buffered silicon using a SSTO buffer layer that acts as a strain-mediating pseudosubstrate and eliminates any structural degradation due to the thermal tension imposed by the Si during cooldown from growth temperature. The prepared BTO thin films are highly crystalline with a pure out-of-plane orientation, exhibiting low coercivity, absence of imprint, good retention, and exceptional fatigue resistance (>10^10^ cycles). Thus, our study enables reliable ferroelectric device operation with large potential benefits for BTO-based CMOS-compatible energy-efficient memory and logic applications.

## Experimental Section

### Sample Preparation and Structural Characterization

All thin films in this study are deposited by pulsed laser deposition using a 248 nm KrF excimer laser on (001)-oriented STO substrates (CrysTec GmbH) and STO-buffered Si substrates (Lumiphase AG). Prior to the deposition process, the STO (001) substrates are treated with buffered hydrofluoric acid and annealed to produce a TiO_2_-termination surface exhibiting atomically smooth terraces. The SSTO buffer layers are grown at 650 °C under an oxygen partial pressure of 0.3 mbar using a laser fluence of 1.43 J cm^–2^ at 2 Hz. The bottom electrode SRO is then deposited at 650 °C with an oxygen partial pressure of 0.015 mbar at the same laser fluence and repetition rate. BTO thin films with varying thicknesses (12–60 nm) are subsequently grown at 650 °C under an oxygen partial pressure of 0.015 mbar at a laser fluence of 1.48 J cm^–2^ at 2 Hz. Finally, the top electrode SRO is deposited under identical growth conditions with the bottom SRO layer. All layers are grown at a target–substrate distance of 50 mm. Film thicknesses are tracked in situ during growth by reflection high-energy electron diffraction and confirmed by *ex situ* X-ray reflectivity measurements.

θ–2θ scans and reciprocal space maps are carried out on a Panalytical X’Pert MRD thin-film diffractometer using a Cu source (CuKα, 1.540598Å) with a 2xGe(220) hybrid monochromator and a PIXcel^3D^ area detector.

### Device Fabrication and Electrical Characterization

All electrical measurements are performed on circular capacitor structures. Top SRO electrode pads with different sizes are fabricated by photolithography (Karl Suss mask aligner MA1006) and ion beam etching (Intlvac Nanoquest Pico 4RF). The dynamic *P*-*E* loops, *I*-*E* curves, PUND measurements, fatigue, and retention measurements are performed on a ferroelectric analyzer (TF analyzer 2000, aixACCT). To investigate the frequency dependence of *P*-*E* loops, triangular waves with frequencies ranging from 0.6 Hz to 5 kHz are applied.

### AFM and PFM Characterization

Atomic force microscopy measurements are performed in tapping mode on a Bruker Dimension Icon scanning probe microscope equipped with Tap300Al-G tips (Budget Sensors, *k* = 40 N m^–1^). Piezoresponse force microscopy measurements are carried out on an Asylum Research Cypher ES scanning probe microscope equipped with PtIr-coated tips (Bruker SCM-PIT-V2, *k* = 3 N m^–1^). PFM measurements are done in air and on-resonance with a tip–sample force of approximately 15 nN.

### Focused Ion Beam (FIB) Sample Preparation

A FEI Helios G5 CX dual-beam SEM-FIB instrument (scanning electron microscope-focused ion beam) was used to prepare electron-transparent samples. Protective EBID carbon (C) and platinum (Pt) layers were deposited on the film before the IBID Pt protective layer. Cross-sectional chunks (dimensions: 14 × 2.0 × 5 μm^3^) were made and transferred using an EasyLift needle to the copper half-grid. Then the sample was initially thinned down to 80–100 nm thickness using standard Ga-beam processing at 30 kV on an opening window with dimensions of 6.0 × 5.0 μm^2^. The remaining chunk is left thick to have a rigid frame to minimize bending and stress release in the e-transparent window. Finally, several low kV cleaning steps (5 and 2 kV) were used to clean the side surfaces of the lamella.

### Scanning Transmission Electron Microscopy (STEM)

The FIB-prepared and loaded TEM grid was transferred immediately to the TEM column using a dedicated low-background double-tilt TEM holder optimized to collect X-rays in the TEM. The microstructure of samples was examined with a double-corrected (probe and image correctors) and monochromated Themis Z scanning transmission electron microscope (Thermo Fisher Scientific) operating at 300 kV. The STEM images were acquired through the HAADF (high-angle annular dark-field) mode. The beam convergence angle was measured to be ∼18 mrad, and the probe current of 30–50 pA was used for STEM imaging. The thin-film heterostructures were imaged in the [100] BTO zone, corresponding to the [110] Si zone.

## Supplementary Material



## Data Availability

The data supporting this study are available in DataverseNL at 10.34894/YMBX4L.

## Notes

Additional structural and electrical measurements along with a table containing a literature comparison of the relevant ferroelectric metrics for BaTiO_3_ thin films grown on silicon substrates (PDF)
