# Supplementary material for: Integration of Imprint-Free and Low Coercivity Ferroelectric BaTiO3 Thin Films on Silicon
Source: Nano Lett. 2026 Jan 8;26(2):764–72. doi: 10.1021/acs.nanolett.5c05139 (PMC12833864; doi:10.1021/acs.nanolett.5c05139)
Supplement: Supplementary file 1 [file nl5c05139_si_001.pdf]

Supplementary Information: Integration of imprint-free and low coercivity ferroelectric $BaTiO_3$ thin films on silicon

Jingtian Zhao,[1,2] Majid Ahmadi,[1,2] Beatriz Noheda,[1,2] and Martin F. Sarott[1,2]

[1] *Zernike Institute for Advanced Materials, University of Groningen, 9747AG Groningen, The Netherlands.*

[2] *Groningen Cognitive Systems and Materials Center (CogniGron), University of Groningen, 9747AG Groningen, The Netherlands.*

(Dated: December 6, 2025)

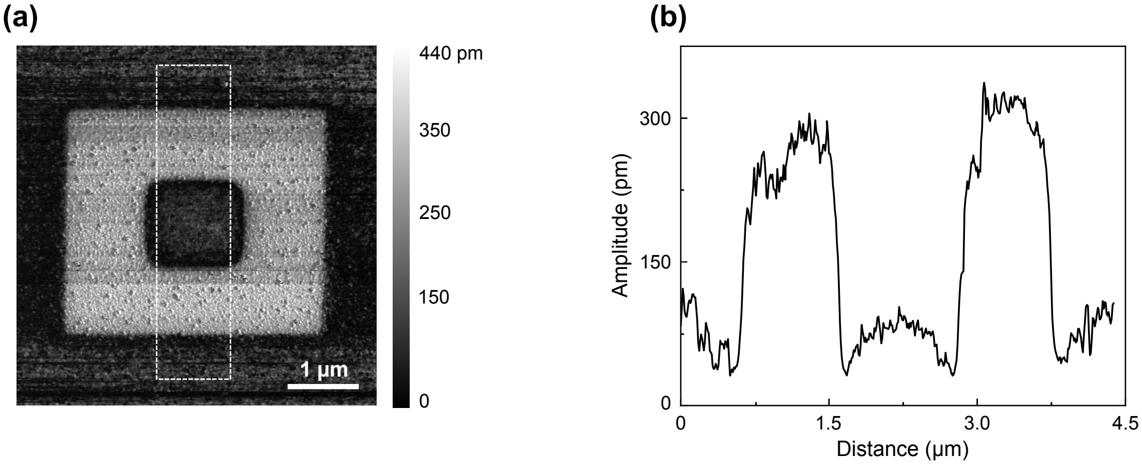

Figure S1. **Vertical PFM amplitude and averaged line profile across the poled area on BTO/SRO/SSTO/STO.** The vertically averaged line profile in (b) across the rectangular dashed outline in (a) shows a clear suppression of the vertical PFM amplitude at the location of the 180° domain wall. The enhanced PFM amplitude of the downward-poled outer box likely originates either from electrostatic charging or a slight misalignment of the laser spot on the back of the PFM cantilever.

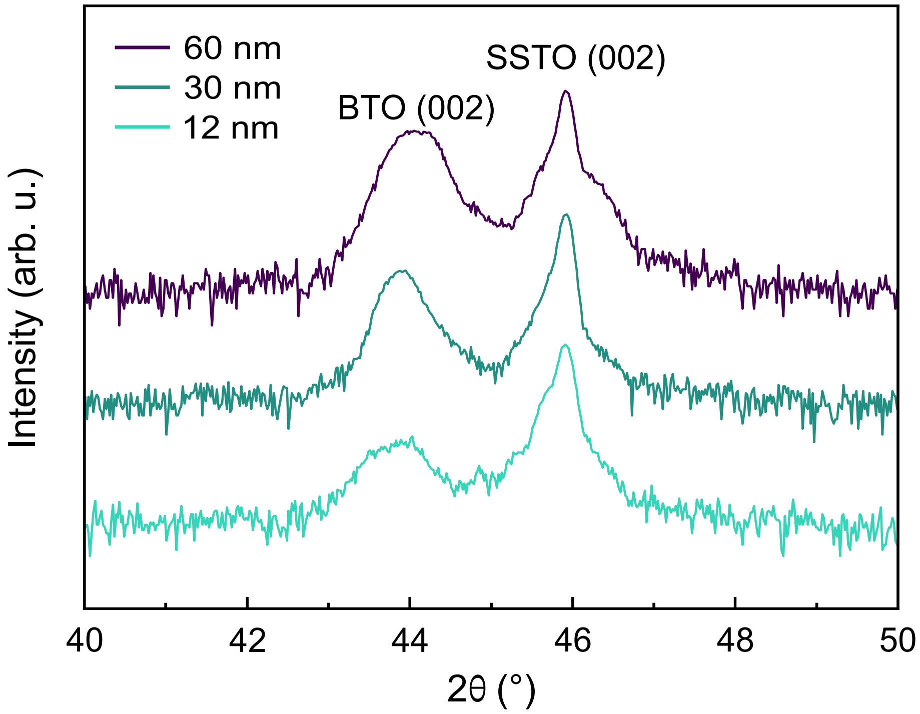

Figure S2. **Symmetric XRD θ–2θ scans of the BTO/SRO/SSTO heterostructure grown on STO-buffered Si** (001) **with different thickness.** For all films, independent of thickness, the BTO tetragonality remains unchanged. We do not observe any deviations from the pure out-of-plane orientation.

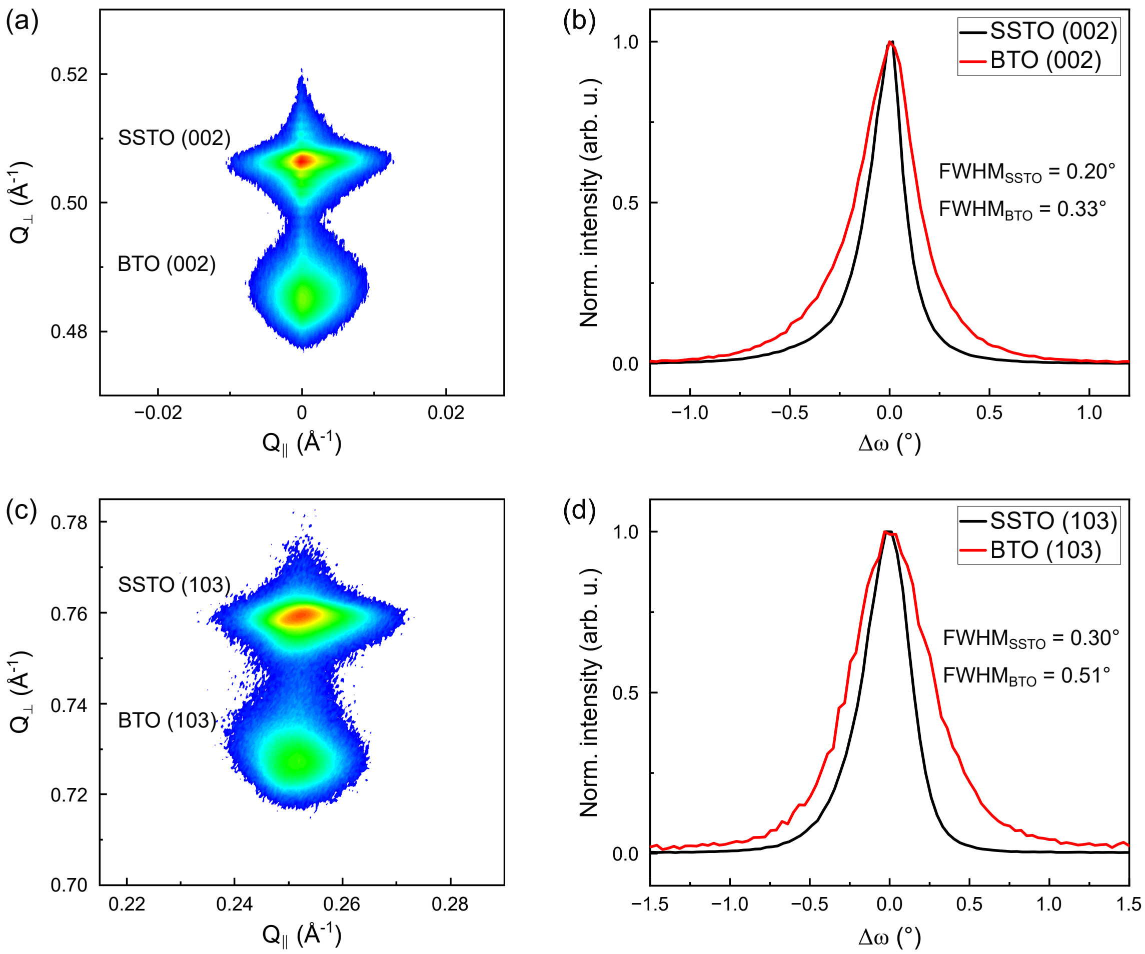

Figure S3. **XRD reciprocal space maps around symmetric and asymmetric BTO reflections, and extracted rocking curves.** (a) RSM around the symmetric BTO 002 peak and (b) extracted rocking curves for the SSTO and BTO 002 peaks. The absence of peak splitting along $Q_\perp$ and butterfly-shaped peak elongations around the BTO reflection, confirm the absence of a multi-a/c-domain configuration and point to a pure c-axis-oriented growth of our BTO on silicon. (c) RSM around the asymmetric BTO 103 peak (d) and extracted rocking curves for the SSTO and BTO 103 reflections.

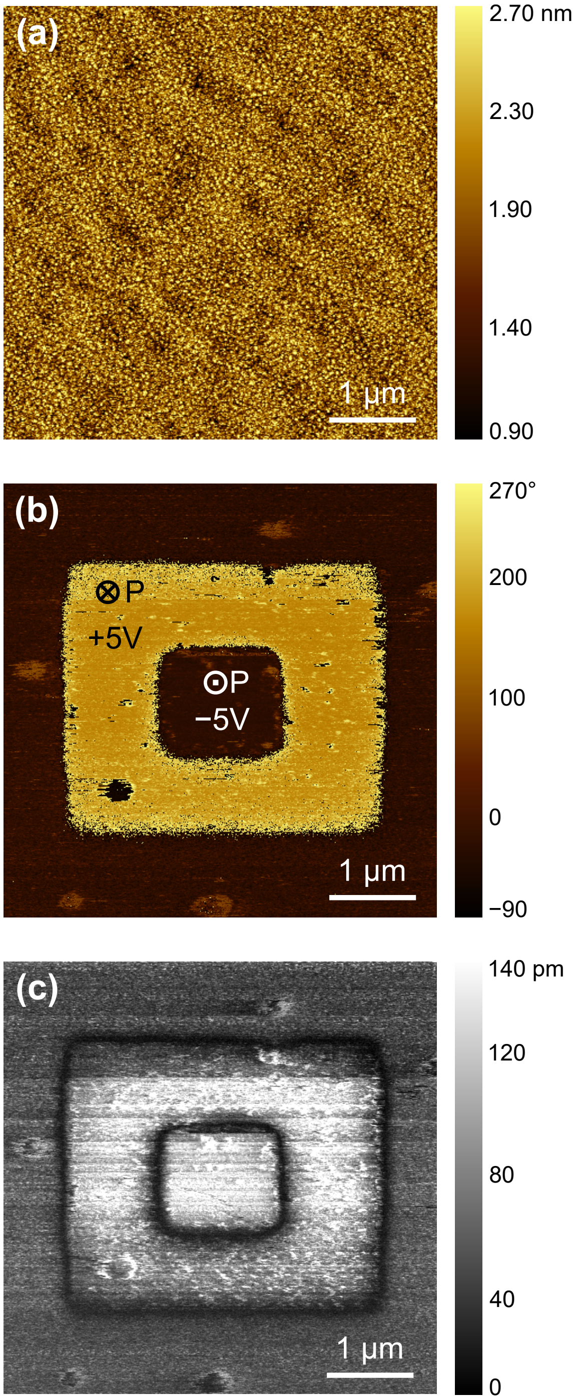

Figure S4. **Surface topography and vertical PFM of the BTO heterostructure on Si.** a) $5{\times}5\,\mu m^2$ atomic force microscopy topographic image. b,c) Vertical PFM phase b) and amplitude c) responses across an electrically poled box-in-box region.

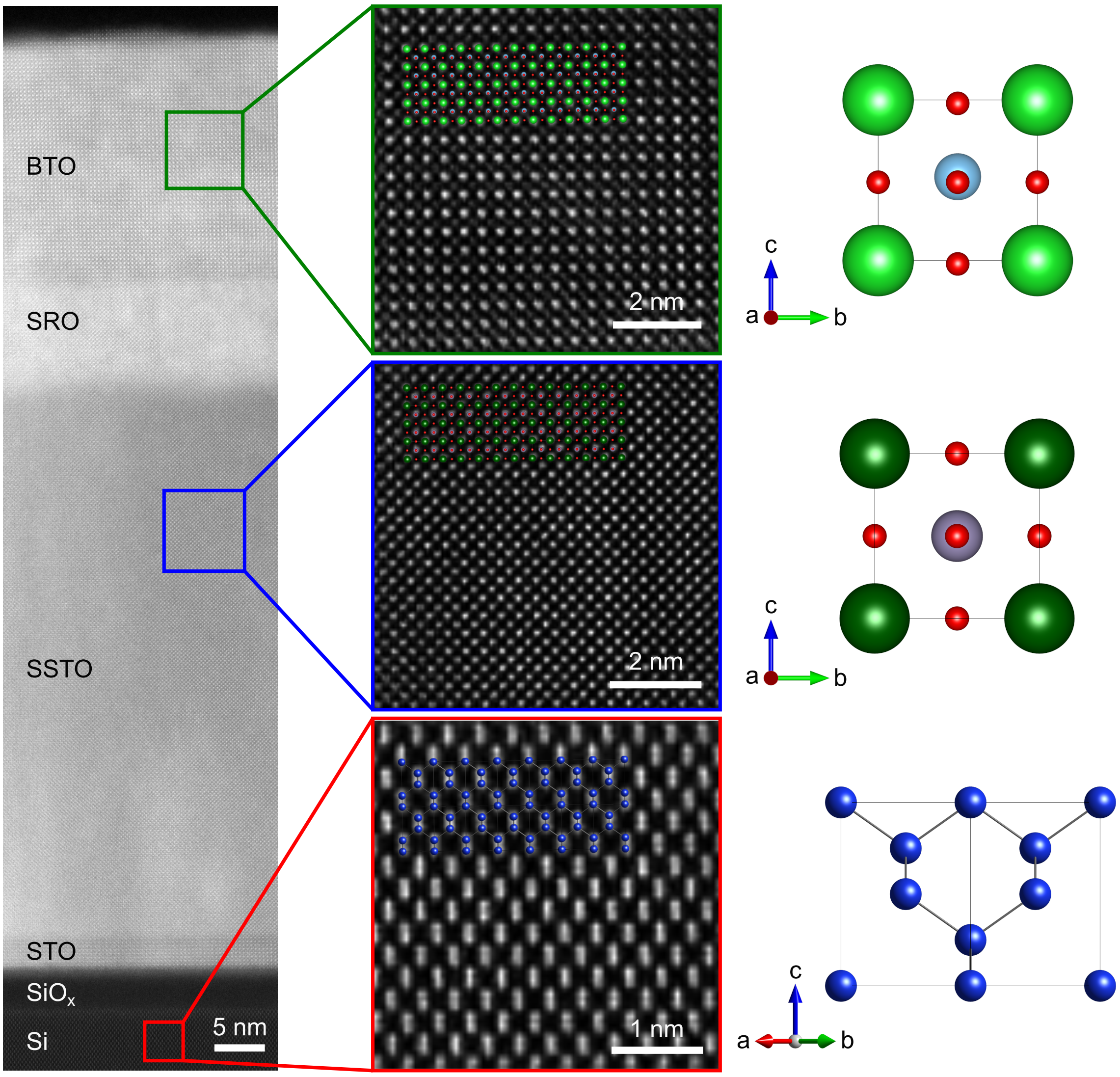

Figure S5. **Atomic resolution structural characterization of the BTO heterostructure on Si.** The cross-sectional HAADF-STEM image of the BTO heterostructure reveals the absence of extended structural defects and chemical interdiffusion between the perovskite layers. The magnified views of the Si, SSTO, and BTO layers, show that all perovskite layers are coherently strained to the SSTO pseudo-substrate. Furthermore, the perovskite unit cells are related to the cubic Si unit cell by a 45° in-plane rotation, corresponding to 45° epitaxy.

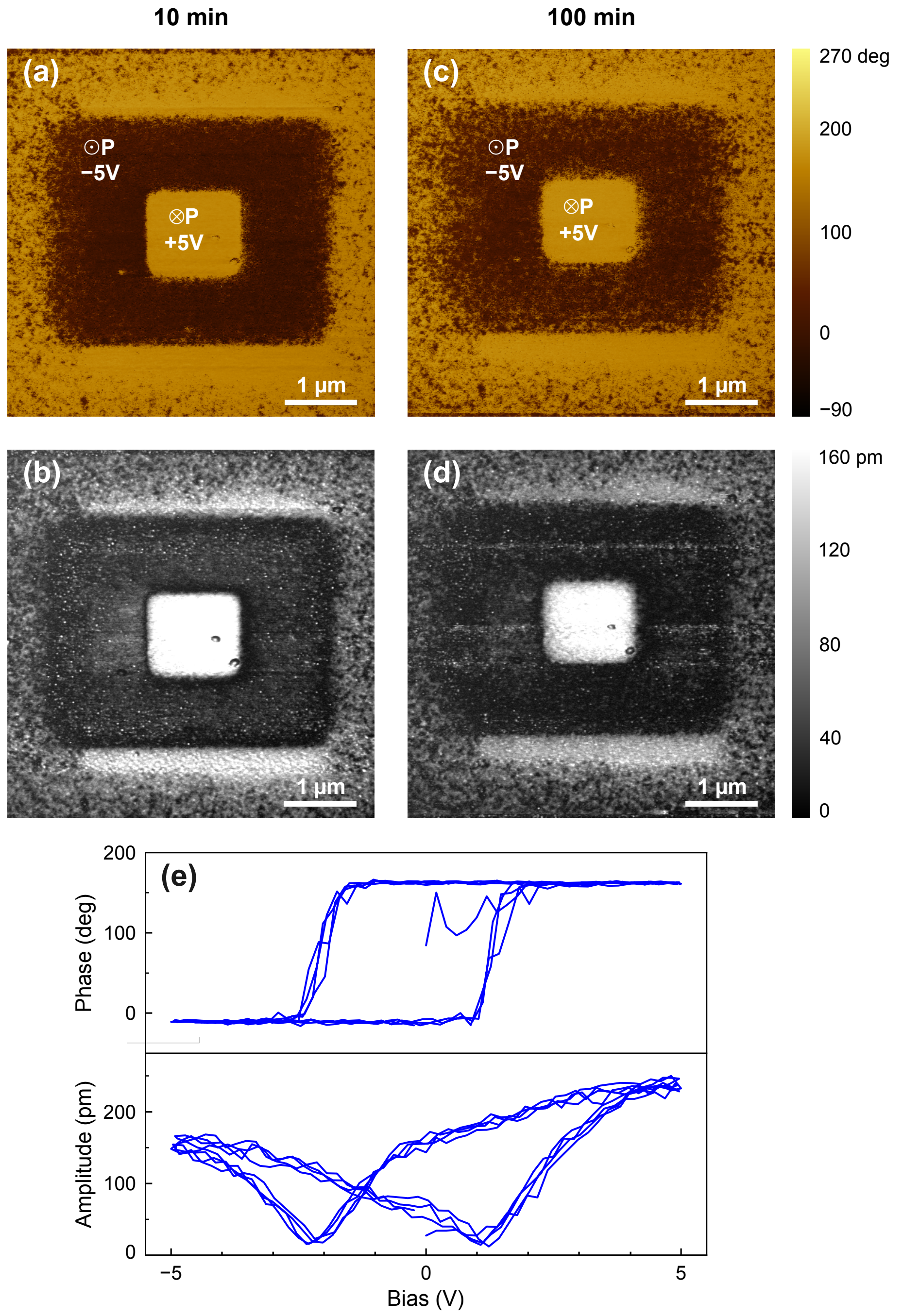

Figure S6. **PFM retention on BTO/SRO/SSTO/STO-Si.** Vertical PFM phase and amplitude (a,b) ∼10 min after box-in-box poling and (c,d) ∼100 min after poling. While a weakening of the amplitude and phase contrast is evident, the poled areas are retained under continuous PFM scanning for at least 100 min. Note that the areas above and below the outer poled exhibit an enhanced amplitude and uniform phase due to a previously applied positive DC voltage in this area. In the pristine state, we observe a multi-domain configuration with a predominate downward-oriented polarization. (e) PFM switching spectroscopy loops on a pristine film area.

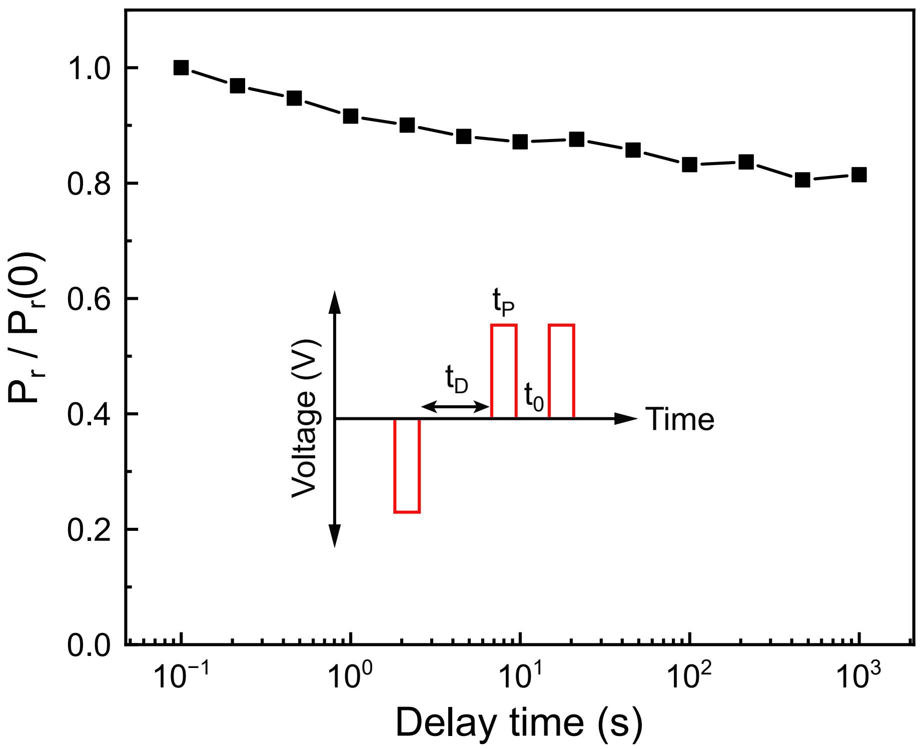

Figure S7. **Device-level polarization retention.** Normalized polarization retention measured on circular BTO capacitors with a diameter of 30 µm. The inset shows a schematic of the applied modified PUND sequence, where the delay time t_D is varied, while both the pulse time $t_p = 250\,\mu s$ and $t_0 = 1\,ms$ are kept constant.

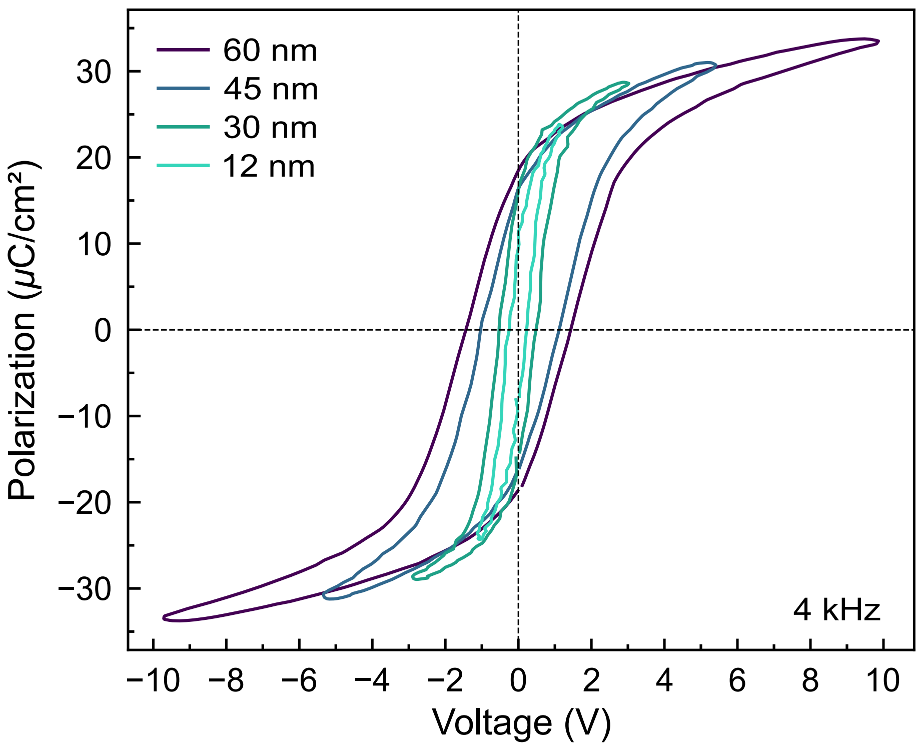

Figure S8. $P\!-\!V$ **profiles corresponding to the** $P\!-\!E$ **loops in Fig. 4c for BTO films of varying thickness.** All $P - V$ profiles are measured at 4 kHz on circular capacitors with an identical diameter of 30 µm. All films are free of imprint and leakage for applied voltages that significantly exceed the coercive field.

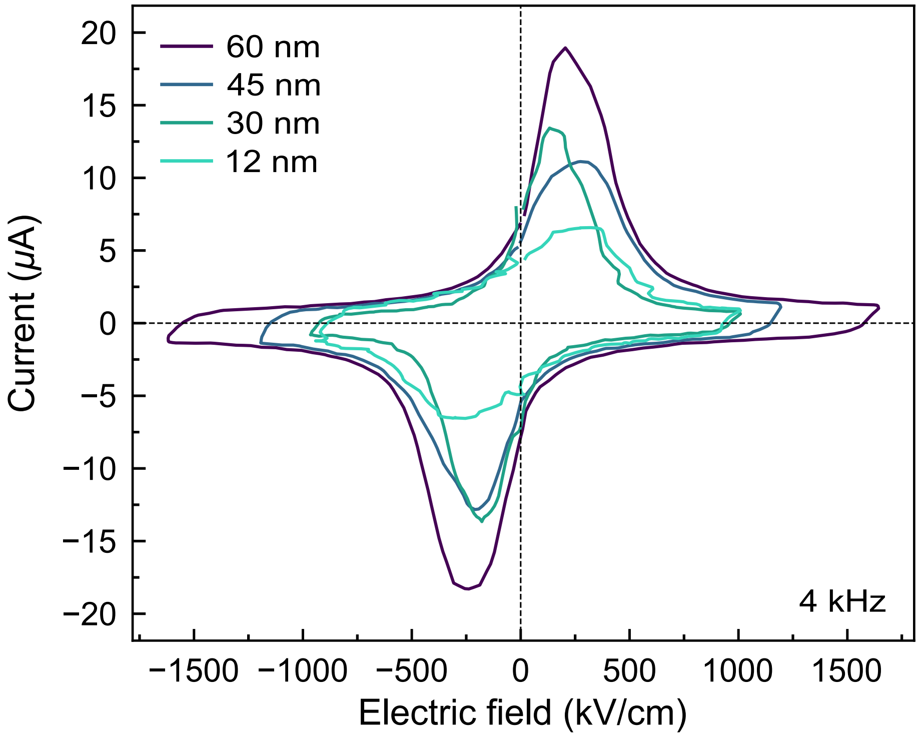

Figure S9. $I-E$ **profiles corresponding to the** $P-E$ **loops in Fig. 4c for BTO films of varying thickness.** All $I-E$ profiles are measured at 4 kHz on circular capacitors with an identical diameter of 30 µm. All films are free of imprint and leakage for applied voltages that significantly exceed the coercive field.

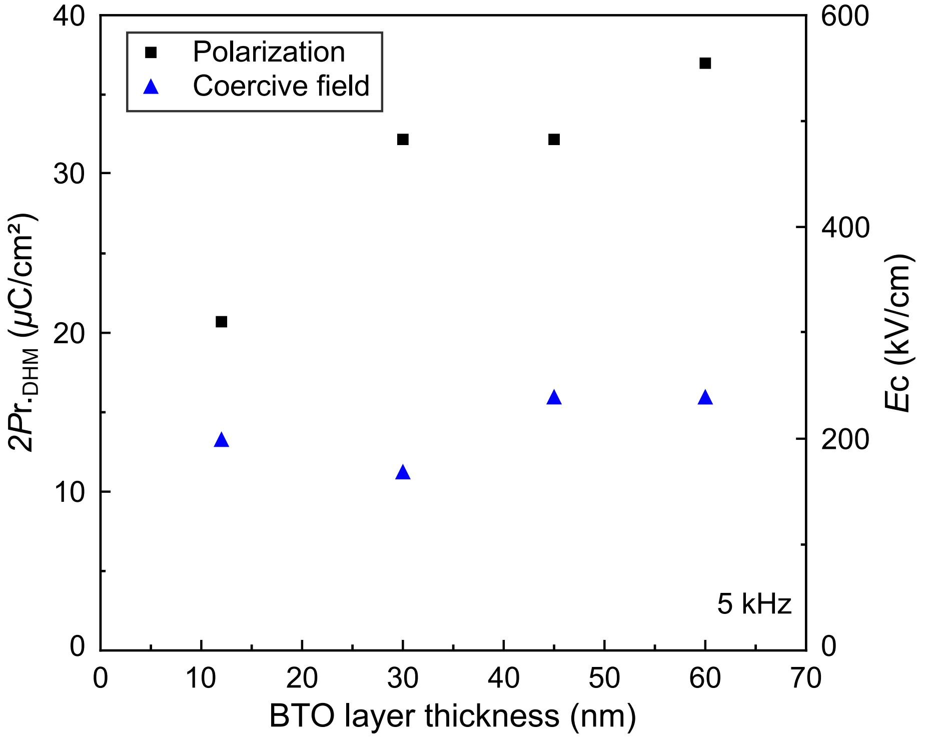

Figure S10. **Thickness dependence of remanent polarization ($2P_\mathrm{r}$) and coercive field (E_c) of BTO films on Si.** With reducing film thickness, we observe a clear reduction of the remanent polarization and an increased depolarization effect. The coercive field, however, remains largely independent of film thickness, implying a reduction of the coercive voltage down to a BTO film thickness of 12 nm.

Table S1. Literature overview of relevant ferroelectric metrics for BTO films grown on Si substrates.

No.	Paper Title	Film Orientation	Thickness (nm)	$2P_r$ ($\mu C\,cm^{-2}$)	E_c ($kV\,cm^{-1}$)	V_c (V)	$V_{imprint}$ (V)	Measurement frequency	Authors
1	Nanoferroelectric domains in ultrathin $BaTiO_3$ films	Polycrystalline	4	0.01	700	0.31	0.05	1 kHz	Drezner *et al.*[1]
2	Origin of compressive strain and phase-transition characteristics of thin $BaTiO_3$ films grown on $LaNiO_3$/Si substrates	Polycrystalline	100	20.40	75.40	0.75	0.22	100 Hz–120 MHz	Qiao *et al.*[2]
3	Ultra-flat $BaTiO_3$ epitaxial films on Si(001) with large out-of-plane polarization	Epitaxial	400	19.13	250.63	1.38	0.22	1−15 kHz	Scigaj *et al.*[3]
4	Coherent epitaxy of a ferroelectric heterostructure on a tri-layered buffer for integration into silicon	Epitaxial	40	34.60	1097	4.39	0.14	1 kHz	Yamada *et al.*[4]
5	Fabrication and ferroelectric properties of $BaTiO_3$ thin films deposited on silicon substrate using PLD	Polycrystalline	200	0.01	157.81	3.16	1.03	10 kHz	Dwivedi *et al.*[5]
6	Control of polar orientation and lattice strain in epitaxial $BaTiO_3$ films on silicon	Epitaxial	103 ± 9	14.96	404 ± 35	4.16	0.21	150 Hz	Lyu *et al.*[6]
7	Enabling ultra-low-voltage switching in $BaTiO_3$	Epitaxial	100	31.05	40.01	0.16	0.18	1 kHz	Jiang *et al.*[7]
8	Achieving high ferroelectric polarization in ultrathin $BaTiO_3$ films on silicon	Polycrystalline	35	31.22	1525.66	8.39	10	5 kHz	Bagul *et al.*[8]
9	This work	Epitaxial	30	25.2	98.59	0.3	0.01 ± 0.03	1 kHz	Zhao *et al.*

[1] Y. Drezner and S. Berger, Nanoferroelectric domains in ultrathin $BaTiO_3$ films. *J. Appl. Phys.* **94**, 6774 (2003).

[2] L. Qiao and X. Bi, Origin of compressive strain and phase transition characteristics of thin $BaTiO_3$ film grown on $LaNiO_3$/Si substrate. *Phys. Status Solidi A* **207**, 2511 (2010).

[3] M. Scigaj, N. Dix, I. Fina, R. Bachelet, B. Warot-Fonrose, J. Fontcuberta, and F. Sánchez, Ultra-flat $BaTiO_3$ epitaxial films on Si(001) with large out-of-plane polarization. *Appl. Phys. Lett.* **102**, 112905 (2013).

[4] H. Yamada, Y. Toyosaki, and A. Sawa, Coherent Epitaxy of a Ferroelectric Heterostructure on a Trilayered Buffer for Integration into Silicon. *Adv. Electron. Mater.* **2**, 1500334 (2016).

[5] V. K. Dwivedi, Fabrication and Ferroelectric Properties of $BaTiO_3$ Thin Films Deposited on Silicon Substrate Using PLD. *Mater. Today Proc.* **5**, 9132 (2018).

[6] J. Lyu, S. Estandía, J. Gazquez, M. F. Chisholm, I. Fina, N. Dix, J. Fontcuberta, and F. Sánchez, Control of Polar Orientation and Lattice Strain in Epitaxial $BaTiO_3$ Films on Silicon. *ACS Appl. Mater. Interfaces* **10**, 25529 (2018).

[7] Y. Jiang, E. Parsonnet, A. Qualls, W. Zhao, S. Susarla, D. Pesquera, A. Dasgupta, M. Acharya, H. Zhang, T. Gosavi, C.-C. Lin, D. E. Nikonov, H. Li, I. A. Young, R. Ramesh, and L. W. Martin, Enabling ultra-low-voltage switching in $BaTiO_3$. *Nat. Mater.* **21**, 779-785 (2022).

[8] P. Bagul, H. Han, P. Lagrain, S. Sergeant, I. Hoflijk, J. Serron, O. Richard, T. Conard, J. Van Houdt, I. De Wolf, and S. R. C. McMitchell, Achieving High Ferroelectric Polarization in Ultrathin $BaTiO_3$ Films on Si. *Adv. Electron. Mater.* **11**, 2400440 (2025).
